# A qualitative study evaluating barriers and enablers to improving antimicrobial use for the management of bacteriuria in hospitalized adults

**DOI:** 10.1017/ash.2024.13

**Published:** 2024-01-31

**Authors:** Emily K. Black, Dianne MacLean, Madison Bell, Heather L. Neville, Olga Kits, Tasha D. Ramsey, Ingrid Sketris, Lynn Johnston

**Affiliations:** 1 Dalhousie University, Halifax, NS, Canada; 2 IWK Health Centre, Halifax, NS, Canada; 3 Nova Scotia Health Authority, Halifax, NS, Canada

## Abstract

**Objective:**

The objective of this study was to explore barriers and enablers to improving the management of bacteriuria in hospitalized adults.

**Design:**

Qualitative study.

**Setting:**

Nova Scotia, Canada.

**Participants:**

Nurses, physicians, and pharmacists involved in the assessment, diagnosis, and treatment of bacteriuria in hospitalized patients.

**Methods:**

Focus groups (FGs) were completed between May and July 2019. FG discussions were facilitated using an interview guide that consisted of open-ended questions coded to the theoretical domains framework (TDF) v2. Discussions were transcribed verbatim then independently coded to the TDFv2 by two members of the research team and compared. Thematic analysis was used to identify themes.

**Results:**

Thirty-three healthcare providers from five hospitals participated (15 pharmacists, 11 nurses, and 7 physicians). The use of antibiotics for the treatment of asymptomatic bacteriuria (ASB) was the main issue identified. Subthemes that related to management of ASB included: “diagnostic uncertainty,” difficulty “ignoring positive urine cultures,” “organizational challenges,” and “how people learn.” Barriers and/or enablers to improving the management of bacteriuria were mapped to 12 theoretical domains within these subthemes. Barriers and enablers identified by participants that were most extensively discussed related to the domains of *environmental context and resources, belief about capabilities*, *social/professional role and identity*, and *social influences*.

**Conclusions:**

Healthcare providers highlighted barriers and recognized enablers that may improve delivery of care to patients with bacteriuria. A wide range of barriers at the individual and organization level to address diagnostic challenges and improve workload should be considered to improve management of bacteriuria.

## Introduction

Antimicrobial resistance has been identified as one of the top global health threats by the World Health Organization.^1^ Patients with infections caused by resistant bacteria have an increased risk of negative outcomes, including death.^2^ It is estimated that by 2050, antimicrobial resistance may be responsible annually for as many as 10 million deaths worldwide if resistance rates continue to rise and,^3^ without further action, as many as 40% of infections in Canada will be antibiotic-resistant.^4^

Urinary tract infections (UTIs) are one of the most common reasons for antibiotic use and one of the most frequent causes of antibiotic-resistant infections in Canada.^4,5^ Antimicrobial use is associated with the development of antimicrobial resistance.^6,7^ As many as half of all patients receive antimicrobial agents inappropriately including patients with asymptomatic bacteriuria (ASB).^8,9,10^ Antimicrobial stewardship efforts are recommended to support appropriate use of antibiotics and preserve effectiveness of these lifesaving medications.^4^

Antimicrobial stewardship has been defined as “coordinated interventions designed to improve and measure the appropriate use of antimicrobial agents by promoting the selection of the optimal antimicrobial drug regimen including dosing, duration of therapy, and route of administration.” Prospective audit and feedback and/or preauthorization are core components of an antimicrobial stewardship program and should be combined with other interventions that meet the unique needs of the institution. Programs should consider implementing interventions that target patients with specific infectious diseases.^11,12^

Suboptimal treatment for UTIs and overprescribing for ASB have been widely reported in the literature.^13,14,15^ As a result, targeted antimicrobial stewardship interventions for patients with UTIs or ASB have been implemented elsewhere. While evidence that supports stewardship interventions has been published, knowledge gaps exist. Most studies evaluating antimicrobial stewardship interventions were completed in large academic centers.^16^ Few studies justify intervention components or incorporate theory into intervention design.^17,18,19^ Finally, the best combination of interventions to improve antimicrobial use for patients with UTIs and ASB that meet the needs of our local population is unknown.

The objective of this study was to explore barriers and enablers to improving the management of bacteriuria in hospitalized adults.

## Methods

### Design and setting

We conducted a qualitative study using the theoretical domains framework, version 2 (TDFv2). The TDFv2 is a set of theoretical constructs designed to assist in developing and successfully implementing interventions.^20,21^ This study was completed in Nova Scotia (NS), Canada at NS Health and approved by the NS Health Research Ethics Board (REB File No. 1024184). NS is a Canadian province with a population of approximately 1 million inhabitants,^22^ and a publicly funded healthcare system^23^ that delivers healthcare services and operates hospitals across the province. NS Health has one multi-campus health sciences center in addition to community hospitals, health centers, and community-based programs across the province.

### Participants

Frontline healthcare providers were recruited to participate in focus groups (FG) to discuss barriers and enablers to effectively manage UTIs and ASB. Nurses (including nurse practitioners and infection prevention and control professionals), physicians, pharmacists, and trainees involved in the assessment, diagnosis, and treatment of UTIs and ASB on hospitalist units were included. Participants, selected based on recommendations from clinical teams at NS Health to ensure representation of multidisciplinary perspectives, were purposively sampled through email communication by a member of the research team from each participating site. Each FG consisted of 5–8 participants. There was at least one FG in each of four management zones of NS Health.^24^

### Data collection

Each FG was facilitated by a trained research coordinator (DM) using an interview guide (Appendix A). The research coordinator is a licensed clinical pharmacist. At the time of data collection, the research coordinator was not employed by NS Health and had no prior relationship with study participants. The research coordinator received training and orientation from a health research methodologist and qualitative researcher team member (OK). Prior to starting the FG discussions, the research coordinator introduced herself (including credentials) and provided an overview of the study goals and objectives. Personal biases or assumptions were not discussed.

The FGs lasted 60–90 minutes. The interview guide consisted of open-ended questions mapped to the TDFv2 to identify barriers and enablers to the appropriate management of UTIs and ASB.^25,26^ Interview questions were piloted in a sample of 5–10 participants and adjusted based on feedback from the pilot phase.

### Analysis

Discussions were transcribed verbatim by the research coordinator (DM) and coded independently by two members of the team (EB, DM). Interviews were analyzed to identify themes and subthemes using the classic analysis strategy by Krueger et al.^27^ Data was mapped to the TDF for analysis.

## Results

Five semi-structured interdisciplinary FGs were conducted with a total of 33 healthcare providers (15 pharmacists, 11 nurses, and 7 physicians) from 2 urban and 3 rural hospitals.

**Figure 1.**
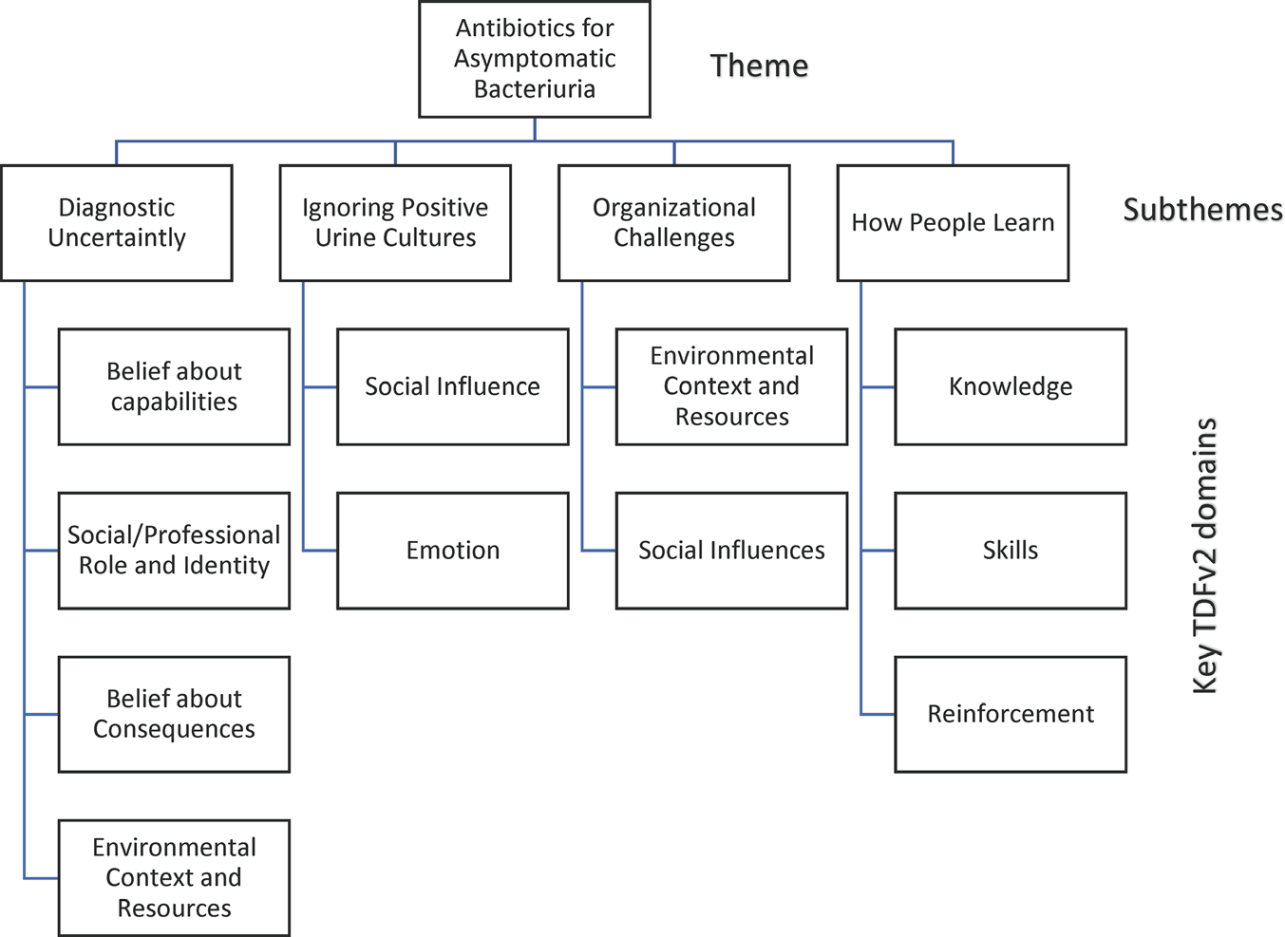
Themes and subthemes linked to the theoretical domains framework, version 2 (TDFv2).

The most extensively discussed challenge relating to management of bacteriuria that emerged as the key theme was inappropriate treatment of ASB, identified by all FGs as an ongoing challenge to appropriate antimicrobial use in hospitalized adults. Subthemes that related to management of ASB included “diagnostic uncertainty,” “ignoring positive urine cultures,” “organizational challenges,” and “how people learn.” Barriers and enablers to improving antimicrobial use for ASB were identified and mapped to 12 of 14 domains in the TDFv2 and grouped under these subthemes (Figure 1). These subthemes predominantly coded to the TDFv2 domains of: *social/professional role and identity*; *belief about capabilities*; *environmental context and resources*; and *social influences*. Examples of barriers coded to the TDFv2 with representative quotes are outlined in Appendix B. Most extensively discussed barriers are discussed below under each subtheme.

### Diagnostic uncertainty

Barriers most extensively discussed by FG participants related to diagnostic uncertainty. Participants highlighted difficulty in confirming a diagnosis of ASB, particularly in older confused patients. Sending urine cultures without an order and/or lack of documentation outlining why urine cultures were sent further complicated the diagnostic process for prescribers. Improved documentation was highlighted as an enabler that may better the diagnostic process and antibiotic use in patients with bacteriuria.

*“But it is hard too, with patients with dementia, they can’t describe their burning because half the time they can’t describe where they are, so you kind of send it [urine culture] because you are like what if it is confusion, what if it is dementia, they could be suffering and actually in pain but not actually able to express it”. (FG 12, Belief about capabilities)*

*“Well sometimes the culture is just sent. It’s not always on the order” (FG 21, Social/Professional Role and Identity)*

*“so a lot of people see one or two of those things [signs or symptoms] and they just do it to rule it out. They see it as a low-cost measure to run urine and see” (FG 15, Belief about Consequences)*

*“Even on call sometimes there is a positive culture, you don’t know the patient, you don’t know why the culture was taken in the first place… Sometimes when it is started on call, the person coming on is not sure why it was started and the communication is not that great.” (FG 31, Environmental Context and Resources)*

*“Maybe having whoever is ordering the culture write down the indication. That would force them to think through why they are doing it.” (FG46, Environmental Context and Resources)*

### Ignoring positive cultures

Ignoring positive urine cultures in asymptomatic patients was also difficult due to healthcare provider and patient/caregiver pressure. Several participants highlighted how fear of missing an infection influences antibiotic prescribing.

*“…or the nursing homes – they are like, oh this is the exact same presentation as what they presented as last time and they were diagnosed with a UTI, so I think they have a UTI” (FG 32, Social Influences)*

*“…if your patient is on antibiotics and you’re like OK, they are covered. If anything happens, they are covered” (FG 13, Emotion)*

### Organizational challenges

Workload created further challenges diagnosing ASB and managing bacteriuria. Busy clinical areas, high patient volumes, rotating staff and locum coverage, and limited personnel was recognized by all groups of healthcare providers included in the FGs.

*“Workload for nurses to have to, for example, if they have to change a catheter to get a proper sample right, that is just another barrier in terms of their time.” (FG46, Environmental Context and Resources)*

*“In the busy environment where we are overworked and all those other things it’s a lot harder to have that conversation with the patient and the family to explain why we are not going to do antibiotics than it is to just give it to them and get them out the door.” (FG11, Environmental Context and Resources and Social Influences)*

*“From a pharmacy point of view on the weekends we don’t have a clinical person up there, so I see a urinalysis pending or I see a result back and I’m like ya (sic) they have bacteria so it must be appropriate, but it is hard to know for sure.” (FG25, Environmental Context and Resources)*

### How people learn

Education and/or reinforcement was generally viewed favorably, although this subtheme overlapped with workload. Some participants reported that workload limited their ability to participate in educational initiatives. Generational differences were also identified by some participants.

*“Pushing education with the staff over the last 10 years, there has been a lot of improvement” (FG 54, Knowledge)*

*“maybe rather than formal education, a poster beside the urine collections bottles – do you have an order. Do your patients actually need this, what are their symptoms. Just reminders that this isn’t necessarily the first line to go to.” (FG33, Reinforcement)*

*“I find the younger crew of doctors coming out now are much more appropriate in management and looking at the whole picture of the UTI. Whereas the older physicians a lot of them will just throw antibiotics when you ask for them. It’s very prevalent. Everybody gets Cipro™ (sic) and they go from there.” (FG 42, Skills)*

Individual participants mentioned other strategies to improve antibiotic use, including audit and feedback and restrictive interventions. Details on additional barriers and enablers discussed and linked to TDFv2 with representative quotes are outlined in Appendix 1.

## Discussion

The FG participants in this study provided important insights into barriers and enablers that exist when treating bacteriuria in hospitalized patients. The most significant challenges related to management of ASB that they identified during FGs were “diagnostic uncertainty,” “ignoring positive cultures,” “organizational challenges,” and “how people learn.”

Our findings are consistent with other studies that have evaluated barriers and enablers to managing infections and, more specifically, ASB. A 2018 study completed by our team explored healthcare providers perceptions on antimicrobial use and stewardship in acute care hospitals in NS. When discussing management of infections in general, many of the identified barriers and enablers closely aligned with those discussed for the management of bacteriuria in this study. Examples of barriers identified included diagnostic uncertainty, patient complexity, and lack of resources due to staffing and time constraints. Education was identified an enabler.^28^ Another study examined barriers specific to managing catheter-associated bacteriuria. Provider’s knowledge of bacteriuria management guidelines and their ability to apply them were identified as the primary barriers to appropriate antimicrobial prescribing.^29^ Other barriers that have been identified in the literature include lack of knowledge, discrepancies between knowledge and practice, and discrepant understandings/gaps in communication between staff, as well as staff and patients.^14,30^

Although discussion of barriers and enablers related to management of infectious diseases including bacteriuria exists in the literature, few studies implement an intervention using evidence-based theory. With UTIs being such a common infection, a high percentage of ASB being inappropriately treated, and rapidly emerging antimicrobial resistance world-wide, promptly and effectively addressing management is critical.^1,2,3,13,14,15^ This study may provide guidance to institutions as an example of how to incorporate theory into design of antimicrobial stewardship interventions. To further support development of an intervention using these results, barriers identified in this study and coded to the TDFv2 can be linked to the COM-B model. COM-B considers capability, opportunity, and motivation as essential components for any behavior change and can be linked to the behavior change wheel to identify interventions for behavior change. The behavior change wheel consists of nine intervention functions to address the behavior and seven types of policy that could be utilized to deliver these intervention functions.^31^

Key barriers identified in this study that coded to the TDFv2 include environmental context/resources, social influences, belief about capabilities, and social/professional role and identity. These domains of the TDFv2 link to physical opportunity, social opportunity, and reflective motivation in the COM-B model. Using the behavior change wheel, potential intervention functions that may address challenges identified include the utilization of training, restriction, environmental restructuring, enablement, modeling, and education and/or persuasion.^31^ We give an example of how we incorporated this theory into designing an evidence based, multifaceted intervention for bacteriuria using the COM-B model and behavior change wheel. Intervention strategies from the behavior change wheel that were ranked highest by our team were audit and feedback, education, clinical orders sets, clinical decision support, and the restrictive intervention of withholding urine culture results. After considering local context and resources, a multifaceted intervention that included audit and feedback and active educational sessions delivered to multidisciplinary teams of healthcare providers was implemented.

There are limitations of this study that are important to consider. A relatively small sample size of healthcare professionals was included, with a smaller proportion of physicians relative to other healthcare providers due to recruitment challenges. We used purposive sampling to ensure a range of perspectives were considered, however, this may have excluded views of individuals who were not invited to participate. All participants were from NS and their opinions may differ from those working outside this province. As previously noted, NS has a publicly funded healthcare system that may present different barriers and enablers when compared to other healthcare funding models. Finally, this study was completed prior to the COVID-19 pandemic. It is likely that resources have been further stretched, organizational challenges have intensified due to the pandemic, and healthcare professionals may be experiencing higher rates of burnout since the time of these FGs. Despite these limitations, our findings highlight specific barriers that are consistent with those previously reported in the literature and provide guidance for the development of management interventions for patients with bacteriuria.

In conclusion, a multifaceted intervention that considers theory and addresses organizational and individual level barriers may be most beneficial in addressing challenges with antimicrobial use for bacteriuria. Local institutions should address challenges with workload to overcome organizational barriers. A decrease in microbiologic testing and individual support for prescribers in the diagnostic process, including enabling interventions such as education and audit and feedback, may further improve antimicrobial use in the hospitalized patient population.

## Supporting information

Black et al. supplementary material 1 Black et al. supplementary material

Black et al. supplementary material 2 Black et al. supplementary material

## Supplementary material

The supplementary material for this article can be found at https://doi.org/10.1017/ash.2024.13.

## Research transparency and reproducibility

To protect confidentiality and privacy of focus group participants, transcripts are not available in a repository.
