# Supplementary material for: A qualitative study evaluating barriers and enablers to improving antimicrobial use for the management of bacteriuria in hospitalized adults
Source: Antimicrob Steward Healthc Epidemiol. 2024 Jan 31;4(1):e17. doi: 10.1017/ash.2024.13 (PMC10897712; doi:10.1017/ash.2024.13)
Supplement: Black et al. supplementary material 1 — Black et al. supplementary material [file S2732494X24000135sup001.docx]

**Interview Guide**

**Optimizing Antimicrobial Stewardship Interventions to Improve Management of Bacteriuria in Hospitalized Adults**

**Questions**

Opening/introduction question

1. Tell us your first name, your role working at the NSHA and a little bit about your practice in general.

**Questions mapped to the Theoretical Domains Framework**

(A) Action – The appropriate management of bacteriuria. Management includes diagnosis, including ordering microbiologic tests for bacteriuria, and treatment (recommending or prescribing pharmacologic or non-pharmacologic therapy)

(C) Context – Within the hospital setting

(T) Time – During hospitalization

(Ta) Target – Adults with bacteriuria

| **Domain (definition) and constructs** | **Interview Question(s)** |
| --- | --- |
| 1. Knowledge: An awareness of the existence of something  - Knowledge (including knowledge of condition/scientific rationale); Procedural knowledge; Knowledge of task environment | The first question aims to gather general information on your experiences caring for patients with urinary tract infections.   1. **Thinking back on your practice over the past year, can you describe the clinical presentation of a typical patient who you might recommend completing a urine culture and/or recommending antibiotics for treatment of urinary tract infections?**   Prompts   - Which aspects of caring for patients with bacteriuria are part of your work as a health care provider? - Is there any additional knowledge or skills (ex. take an accurate patient history, complete a physical exam, properly collect clean catch midstream urine sample, synthesize information to make a diagnosis) you need to care for patients with bacteriuria in your practice? |
| 1. Skills: An ability or proficiency acquired through practice  - Skills; skill development; interpersonal skills; skill assessment; competence; ability; practice |  |
| 1. Social/Professional Role and Identity: A coherent set of behaviours and displayed personal qualities of an individual in a social or work setting  - Professional identity and role; Social identity; Professional boundaries; Professional confidence; Group identity; Leadership; Organizational commitment |  |
| 1. Beliefs about Capabilities: Acceptance of the truth, reality, or validity about an ability, talent, or facility that a person can put to constructive use  - Self-confidence; Perceived competence; Self-efficacy; Perceived behavioural control; Beliefs; Self-esteem and confidence; Empowerment | 1. **How will improvement in management of hospitalized adults with bacteriuria be a benefit to patients and the public in general?**   Prompts   - How confident are you that you can appropriately manage bacteriuria in hospitalized adults? (Ex. avoid antibiotics for positive urine culture results in asymptomatic patients with bacteriuria when antibiotics are not indicated) - Are you optimistic that management of urinary tract infections in hospitalized adults can be improved? Why or why not? |
| 1. Optimism: The confidence that things will happen for the best or that desired goals will be attained  - Optimism; Pessimism; Unrealistic optimism; Identity |  |
| 1. Beliefs about Consequences: Acceptance of the truth, reality, or validity about outcomes of behaviour in a given situation  - Beliefs; Outcome expectations; Characteristics of outcome expectations; Anticipated regret; Consequences |  |
| 1. Reinforcement: Increasing the probability of a response by arranging a dependent relationship, or contingency between the response and a given stimulus  - Rewards; Incentives; Punishments; Consequences; Reinforcement; Contingencies; Sanctions | 1. **Is there an initiative you can think of that may improve your ability to do your job caring for patients with bacteriuria?**   Prompt   - An example of an initiative is making urine culture requisition forms less accessible - Would incentives or disincentives make you more likely to appropriately manage bacteriuria |
| 1. Intentions: A conscious decision to perform a behaviour or a resolve to act in a certain way  - Stability of intentions; Stages of change model; Trans theoretical model and stages of change | 1. **Compared to other conditions you encounter on your floor or unit, how important is it for you to manage bacteriuria?**   Prompt   - Examples of conditions may include falls or hospital acquired pneumonia |
| 1. Goals: Mental representation of outcomes or end states that an individual wants to achieve   Goals; goal priority; goal/target setting; action planning; implementation intention |  |
| 1. Memory, attention, and decision processes: The ability to retain information, focus selectively on aspects of the environment and choose between two or more alternatives   Memory; Attention; Attention control; Decision making; Cognitive overload/tiredness | 1. **Can you describe how physical or resource factors facilitate or hinder your ability to appropriately manage hospitalized adults with bacteriuria?**   Prompts  - Are there times when there are too many distractions that limit your ability to apply evidence based management for urinary tract infections in hospitalized adults?  - What factors within your clinical environment facilitate or hinder your ability to appropriately manage hospitalized adults with bacteriuria? Examples of internal factors include access to computers on the unit  - What factors outside your clinical environment facilitate or hinder your ability to appropriately manage hospitalized adults with bacteriuria. Examples external factors include access to patient information (including diagnostic information, labs, or medication use), information technology, or human resources. |
| 1. Environmental Context and Resources: Any circumstances of a person’s situation or environment that discourages or encourages the development of skills and abilities, independence, social competence, and adaptive behaviour   Environmental stressors; Resources / material resources; Organizational culture/climate; Salient events/critical incidents; Person x environment interaction  Barriers and facilitators |  |
| 1. Social influences: Those interpersonal processes that can cause individuals to change their thoughts, feelings, or behaviours   Social pressure or norms; Group conformity; Social comparisons; Group norms; Social support; Power; Intergroup conflict; Alienation; Group identity; Modelling | 1. **Can you describe any social influences that facilitate or hinder your ability to appropriately manage hospitalized adults with bacteriuria?**   Prompts  - What impact does other healthcare providers or external programs/initiatives (Ex. Choosing Wisely Canada) influence your decision on how to manage bacteriuria in hospitalized adults?  - To what extent does diagnosis or prescribing of antimicrobial agents contribute to fear, anxiety, or stress in you?  - How do patients’ (or family members) emotions or influence affect your decisions on how to manage bacteriuria in hospitalized adults? |
| 1. Emotion: A complex reaction pattern, involving experiential, behavioural, and physiological elements, by which the individual attempts to deal with a personally significant matter or event   Fear; Anxiety; Affect; Stress; Depression; Positive/negative affect; Burn-out |  |
| 1. Behavioural Regulations: Anything aimed at managing or changing objectively observed or measured actions   Self-monitoring; Breaking habit; Action planning | 1. **Would audit and feedback help monitor whether your patients are appropriately treated?**   Prompt  Audit and feedback refers to a measure of performance (or audit) compared to professional standards. Results are feedback to the individual or group. (https://www.cochrane.org/CD000259/EPOC_audit-and-feedback-effects-on-professional-practice-and-patient-outcomes)   - Would you prefer individual level feedback or floor/unit level feedback? - Would you prefer feedback to be provided verbally, electronically (ex. Email), or paper-based? |

**Is there anything else you would like to share with us today about barriers and facilitators to managing urinary tract infections and asymptomatic bacteriuria that we have not addressed?**

References

Huijg JM, Gebhardt WA, Crone MR, Dusseldorp E, Presseau J. Discriminant content validity of a theoretical domains framework questionnaire for use in implementation research. Implementation Science 2014;9:11.

Curran JA, Brehaut J, Patey AM, Osmond M, Stiell I, Grimshaw JM. Understanding the Canadian adult CT head rule trial: use of the theoretical domains framework for process evaluation. Implementation Science 2013;8:25.
