# Supplementary material for: A qualitative study evaluating barriers and enablers to improving antimicrobial use for the management of bacteriuria in hospitalized adults
Source: Antimicrob Steward Healthc Epidemiol. 2024 Jan 31;4(1):e17. doi: 10.1017/ash.2024.13 (PMC10897712; doi:10.1017/ash.2024.13)
Supplement: Black et al. supplementary material 2 — Black et al. supplementary material [file S2732494X24000135sup002.docx]

**Appendix A: Coding to the Theoretical Domains Framework with representative quotes**

| **Domain (definition)** | **Coding Guide** | **Representative quote** |
| --- | --- | --- |
| 1. Knowledge   (An awareness of the existence of something)   - Knowledge (including knowledge of condition/scientific rationale) - Procedural knowledge - Knowledge of task environment | **Do you know about X?**  Knowledge of guidelines (treatment ASB and UTIs)  Knowledge of when to order culture  Knowledge of when catheters are indicated  Awareness of consequences of antimicrobial use (ex. ADRs, cost, AMR)  Knowledge educational resources exist (ex. Guidelines, PPOs)  Knowledge of what defines ASB/UTI | “It is just the families lack of knowledge and they are demanding for treatment” (FG 48)  “definitely education for the nurses as well, because their first response is to send urines” (FG 42)  “pushing education with the staff over the last 10 years there has been a lot of improvement” (FG 54) |
| 1. Skills   (An ability or proficiency acquired through practice)   - Skills, Skill development, interpersonal skills, skill assessment - Competence, Ability, Practice | **Do you know how to do X?**  Ability or competence in changing catheters  Ability to properly collect urine from catheterized patients  Skills to communicate with patients  Ability to accurately diagnosis ASB/UTIs  Ability to treat patients with bacteriuria | “I find the younger crew of doctors coming out now are much more appropriate in management and looking at the whole picture of the UTI. Whereas the older physicians, a lot of them will just throw antibiotics when you ask for them. It’s very prevalent. Everybody gets Cipro and they go from there.” (FG 42)  “I would never send a culture from a catheter. I would always change the catheter before sending a culture. Which doesn’t happen sometimes. But you have to write – change catheter then send culture.” (FG 52)  “I have had some nurses say oh I have never taken it from the catheter bag, I took it from the catheter tubing” (FG 52)  “So we are collecting it [urine samples] from not necessarily sterile things to begin with” (FG 12)  “we are taking away the symptom and treating what is on paper not the symptoms” (FG 14) |
| 1. Social/Professional Role and Identity   (A coherent set of behaviours and displayed personal qualities of an individual in a social or work setting)   - Professional identity and role - Social identity - Professional boundaries - Professional confidence - Group identity - Leadership - Organizational commitment | **Is doing X compatible or in conflict with standards/identity?**  Professional role in ordering cultures  Understanding and negotiating professional roles | “the problem is you send it, who is going to deal with it. Without the order. Do you know what I mean? It’s actually been in the courts. It’s been a whole issue, over a patient, it was sent, they died. And there was no order for it. That was many years ago. And the thing is ,who is going to deal with it if you just send it” (FG 45)  “I think it is a mindset if they see symptoms they want it back because it takes a few days, so when they bring it to the physician they are like – oh I already did this for you so you don’t have to order it, wait, then order antibiotics. I think they are trying to make the process faster for the physician” (FG 12)  “but that is something that came up when I was educating nurses, you know – try not to send a UA without a physician order because that is something that I was receiving feedback from physicians about, is like sometimes the UA is already sent and I haven’t even ordered it. But I would imagine if you have an established relationship with the physician you feel comfortable sending it before having their OK, that you would probably say I will get their OK later. But I did hear from both nursing and physician staff that they definitely get sent without a physician order” (FG 14)  “Well sometimes the culture is just sent. Its not always on the order” (FG 21)  “Like when we started upstairs I remember I would come in the morning and they would say oh there is a urine culture back because someone had dark urine and it was just sent by staff overnight. Like that was happening pretty frequently a decade ago and that doesn’t happen anymore. Or very rarely. (FG 54) |
| 1. Beliefs about Capabilities   (Acceptance of the truth, reality, or validity about an ability, talent, or facility that a person can put to constructive use)   - Self-confidence - Perceived competence - Self-efficacy - Perceived behavioural control - Beliefs - Self-esteem and confidence - Empowerment | **How difficult or easy is it for you to do X?**  Confidence or belief one’s ability to diagnose UTIs (challenges in confused/delirious patients, “not quite sure”)  Confidence or belief in one’s ability to ignore positive urine cultures  Confidence or belief in one’s ability to stop an antibiotic once started by others  Confidence in dealing with pressure to diagnosis or prescribe antibiotics for ASB/UTIs  Confidence in making decisions about whether or not to prescribe for UTIs | “there are often cultures that come back positive and we continue treatment for a week or 10 days, or longer, cuz they are improving. But are they improving because of the antibiotic or are they improving because we are giving them a whole bunch of fluid every time we give them an antibiotic. It is hard to say. At that point do we continue treating or do we stop it?” (FG33)  “But it is hard too, with patients with dementia, they can’t describe their burning because half the time they can’t describe where they are, so you kind of send it because you are like what if it is confusion, what if it is dementia, they could be suffering and actually in pain but not actually able to express it”. (FG 12)  “I think confusion is the really tricky one because when patients are confused or loved ones are confused ,people are like grasping at straws to find some kind of cause for it. And so it would be really hard to talk a loved one out of treating a UTI, that’s not really a UTI, but they think it could help someone’s confusion. Cause I found myself in that situation lots of times and if there is actual bacteria showing, whether its colonized or not, it would be very hard to talk someone out of reacting or treating that.” (FG 14)  “I feel like foul smelling urine and increase confusion are probably the two most common reasons why nursing staff will send a urine culture. And it is hard especially if you have a patient that is already incompetent and you can’t determine their baseline confusion. You can’t always tell if they are having increased confusion and their only symptom is maybe the fact that they have very foul smelling urine. Because they are not able to tell you any symptoms. (FG 27)  “Even on call sometime there is a positive culture ,you don’t know the patient, you don’t know why the culture was taken in the first place...” (FG 31)  “If you have a confused elderly patient, they can’t just report these symptoms to you, but if they come in febrile, borderline septic, chest x-ray doesn’t show anything sometimes you are going to put them on prophylactically just in case” (FG 42)  “from my point of view I’ve got my peers giving the initial diagnosis, and maybe there is sometimes some reluctance to reverse your colleagues decisions. To tell families – oh my colleague was wrong about this. Certain pressures like that.” (FG 46)  “The challenge we get into upstairs is that people are often so sick and frail that they often present with fever. And so then part of that fever and infectious work up often involves a urine culture. Almost automatically. And so then you are in a situation where someone has a chest xray that shows pneumonia but now they also have a positive urine culture and it is hard to determine -what if the bacteria that is on the urine culture is not covered by what you are thinking of putting them on as an antimicrobial for the chest. And so then you are in the situation where they may be treated for the asymptomatic bacteriuria, but in a person with a fever with 2 potential sources of infection if they are delirious and you are not sure about they symptoms, it is hard to tease that out.” (FG 54) |
| 1. Optimism   (The confidence that things will happen for the best or that desired goals will be attained)   - Optimism - Pessimism - Unrealistic optimism - Identity | **How confident are you that the problem of implementing X will be solved?**  Optimism about the outcomes of a particular intervention (ex. Withholding cultures had or will have good results) | “when we started upstairs I remember I would come in the morning and they would say oh there is a urine culture back because someone had dark urine and it was just sent by the staff overnight. Like that was happening pretty frequently a decade ago and that doesn’t happen anymore. Or very rarely. I would think depending on the floor.” (FG 54) |
| 1. Beliefs about Consequences   (Acceptance of the truth, reality, or validity about outcomes of behaviour in a given situation)   - Beliefs - Outcome expectations - Characteristics of outcome expectations - Anticipated regret - Consequences | **What do you think will happen if you do X?**  Consequence, belief, or expected outcome of missing a diagnosis (UTI or other condition)  Consequences or beliefs about of antibiotic use (risks of ADRs, AMR, etc…)  Consequences or beliefs of using catheters  Belief that recommendations or outcomes are different for “their type of patients” | “so a lot of people see one or two of those things and then they just do it to rule it out. They see it as a low cost measure to run urine and see” (FG 15)  “we are not thinking about the long term effects of putting them on antibiotics, the long term effects of what that could mean for them to stay longer in the hospital or anything like that. Or what it could mean for their morbidity in the hospital.” (FG 13)  “I think a lot of people think – oh just my loved one or just one more person, give them the antibiotics and yes maybe antimicrobial resistance is a big upcoming thing that is happening, and it is happening right now but what is just one more prescription going to do.” (FG 13)  “if you watch someone that comes in with frequent UTI’s, if you look at their cultures over several years you can watch it go from pan sensitive E.coli to only sensitive to meropenem. And I have seen that.” (FG 22)  “from my point of view, there is always a thing in the back of my head, if I treat someone with an antibiotic, and they get C. diff and get very sick, I am less likely to get in trouble for that from a family for example, than if I didn’t treat them and they got sick from an unrelated issue and then the family blames me for not treating a quote, unquote UTI. So there is that issue if I don’t do what the patient and family expects the patient has a bad outcome, even if I know what I am doing is correct. You know. Am I going to be held responsible? Whereas if I harm the patient with antibiotics, I am not going to get in trouble for that. That always plays in the back of your mind with every decision.” (FG 46) |
| 1. Reinforcement   (Increasing the probability of a response by arranging a dependent relationship, or contingency between the response and a given stimulus)   - Rewards, Incentives - Punishment, Consequences - Reinforcement - Contingencies - Sanctions | **Are there incentives to do X?**  Reinforcement to families (no need for antibiotics, close monitoring)  Past experience reinforces or influences decisions, challenges in changing mindset, culture  Reminders (ex. signage, PPO, checklist, algorithm)  Require criteria for order to be sent | “sometimes their own experience too. That one patient, that one time that they didn’t treat, that went septic, and now they want to cover their base every time” (FG 16)  “I like the idea of the little box with the reason for the urinalysis, just a phrase, just like on a chest X-ray. I think that would be quality care.” (FG 28)  “maybe rather than formal education, a poster beside the urine collections bottles - “do you have an order”. ‘Do your patients actually need this, what are their symptoms”. Just reminders that this isn’t necessarily the first line to go to.” (FG 33) |
| 1. Intentions   (A conscious decision to perform a behaviour or a resolve to act in a certain way)   - Stability of intentions - Stages of change model - Trans theoretical model and stages of change | **Have they made a decision to do X?**  Intentionally not ordering a urine culture if asymptomatic  Intentionally deciding to include antimicrobial duration on every prescription order  Intentionally using catheters despite recognition, they are not indicated  Intentionally choosing to use antibiotics (or delay/avoid antibiotics) | “even though there is the - what is it called – the CAUTI (sic) recommendations Catheter associated urinary tract infections, a big document that says, when you should be using a catheter and when you shouldn’t be using a catheter, probably 50-75% of the time we are putting catheters in patients that really shouldn’t have catheters put in**.”** (FG 13)  “usually in the middle of the night , 2 o’clock and they are up 5 times within a hour, we will send a urinalysis” (FG 24)  “I try not to order it unless they are symptomatic because then you are wondering if you should treat.” (FG 31)  we do try to avoid catheters at all, we do, and if they have them we try to reassess them and get them, take out as quickly as possible. So we do try and instill that practice. (FG 45) |
| 1. Goals   (Mental representation of outcomes or end states that an individual wants to achieve)   - Goals, goal priority, goal/target setting, action planning, implementation intention | **How much do they want to do X?**  Treat bacteriuria so we can get the patient discharged  Priority of caring for patients with bacteriuria (High priority or low)  Goal or priority to learn more or acquire knowledge about management of bacteriuria (improving patient care vs just some module you have to do)  Priority to contribute to reducing AMR  Belief that AMR is not an important priority | “give them and get them out the door.” (Truro, FG 11)  “one of the goals of hospital care is to get the catheter out as soon as you can.” (FG 23)  “we can get them home if the family is willing to take them. So to us, the faster we can treat somebody whether they need it or not is the problem.” (FG 15) |
| 1. Memory, attention, and decision processes   (The ability to retain information, focus selectively on aspects of the environment and choose between two or more alternatives)   - Memory - Attention - Attention control - Decision making - Cognitive overload/tiredness | **Is X something you usually do?**  Cognitive overload due to being too busy, too high-volume patients  Many educational initiatives, too much information  Remembering specific patient details  Alert fatigue | “anything that triggers a renewed reminder. As opposed to just passing that wall every day that has the giant sign that is blocked by a bed or a linen cart or something.” (FG53)  “I find that just everyone is being bombarded with knowledge of things they need to know. Especially new nurses that come and you can relate to this, it is so much information. What is important? Everything is important. To them. Half of it is mandatory and they can’t even get that much in because it is just so much information about everything.” (FG13) |
| 1. Environmental Context and Resources   (Any circumstances of a person’s situation or environment that discourages or encourages the development of skills and abilities, independence, social competence, and adaptive behaviour)   - Environmental stressors - Resources / material resources - Organizational culture/climate - Salient events/critical incidents - Person x environment interaction - Barriers and facilitators | **To what extend do physical or resource factors facilitate or hinder X?**  Getting patient information from community or other institutional settings (including access to EMR, SHARE etc…)  Documentation  Time saving to have catheters in (time to take patients to bathroom)  Time (general) and staffing, overworked, busy environments  Paper vs electronic records  Need for hospital beds, overcrowding  Educational initiatives (presentations, posters) suggested to improve knowledge and awareness  Checklists, algorithms, pre-printed orders, guidelines, policies  Handing over patients or going off on weekends, weekend coverage, different nurse coverage, transfers from the emergency department | Workload  “UTI on-line module that anyone can go on and do but again it is a time resource issue. Has everyone done it? I haven’t even done it and I have a keen interest in UTI and I haven’t even looked at it.” (FG14)  “we’re so busy, we don’t have time to check our emails” (FG12)  “in the busy environment where we are overworked and all those other things it’s a lot harder to have that conversation with the patient and the family to explain why we are not going to do antibiotics than it is to just give it to them and get them out the door.” (FG11)  “from a pharmacy point of view on the weekends we don’t have a clinical person up there, so I see a urinalysis pending or I see a result back and I’m like ya they have bacteria so it must be appropriate but it is hard to know for sure. And even sometimes when you go to the chart the steps that are there to look for the symptoms you don’t necessary find it” (FG25)  “people do just get them automatically in emerg. I would say more that 50% of my patients that are frail and elderly, I will go in and see the day after admission and they had a catheter placed in emerg because they weren’t very mobile. So from that sort of preventative, sort of thing would be good. And I think prevention in the emergency room on admission would be good, so we can only control what is happening on our unit we can’t really control as a group what is happening before people get to us” (FG54)  “workload for nurses to have to, for example, if they have to change a catheter to get a proper sample right, that is just another barrier in terms of their time.” (FG46)  “bacteria in the urine, I think the first thing that jumps to the nurses head is – that is something we need to treat, we need to get it fixed so we can get them home. It is one more thing that is going to keep them here and that is where everyone’s head is always at. Just we are just trying to get them fixed and get them out and get the next person in because we are so busy.” (FG13)  “I feel like it is overworked physicians too. They don’t have time to fully, properly assess whether they should.” (FG12)  “the changing of nurses - sometimes it doesn’t get re-picked up and the physicians are so busy so it doesn’t get re-picked up and so they are on antibiotics for a week when really their symptoms haven’t changed or they are not showing the symptoms they were showing that one day anymore” (FG12)  “it easier to have the catheter in for the nurses. Instead of – I don’t actually have to get hands on and help them walk to the bathroom. So I think that sometimes the catheter might be seen as something that is easier or time saving in terms of nursing resources.” (FG52)  “from a pharmacy point of view on the weekends we don’t have a clinical person up there, so I see a urinalysis pending or I see a result back and I’m like ya they have bacteria so it must be appropriate, but it is hard to know for sure. And even sometimes when you go to the chart the steps that are there to look for the symptoms you don’t necessary find it.” (FG25)  Documentation  “very occasionally someone will be admitted through the emergency room that has an antibiotic order with no stop date, there has been the odd time I have picked up the chart to discharge the person and realize they have been getting an antibiotic for 10, 12, 13 days unbeknownst to me because the handover was a patient, we are going to give them amox for 5 days but the orders ticked off, but then as a physician, we, if we look at that order great, if we don’t see the order because we didn’t write it and we don’t have to co-sign that order unless we are physically finding the MAR every day and review the MAR, and you can imagine an inpatient ward, that is sometimes difficult…. It is just, sometimes things like that can get missed. So I think if there was, I think it would be nice to have sort of a uniform approach to say whenever there is an antimicrobial order there is a duration attached to an antimicrobial I think that would be a great suggestion.” (FG54)  “I think if someone is ordering a urinalysis or a urine culture it should have to say a rationale why. Like are they symptomatic, is it because they have UTI symptoms, is it because there is confusion, or is it because maybe foul smelling urine or something like that. If they indicate why, then when the results come back, if we can look back at that it would be helpful, I think for my practice.” (FG33)  “maybe having whoever is ordering the culture maybe would have to write down the indication and that would force them to think through why they are doing it.” (FG46)  “I write it [urine culture] on a piece of paper, give it to one person, they enter it into the computer, put the physical piece of paper in the chart back somewhere, then the nurse will eventually find it, somehow.” (FG46)  “one of the things that I learned once I started is that certain places in community are offering blood tests, and urines and those report to the pharmacy system but they do not report to the clinical portal.” (FG33)  “a list of criteria that you could check off and say OK if this is happening then we can try changing the catheter and then, next step would be a UA, C&S” (FG22)  “Even on call sometimes there is a positive culture, you don’t know the patient, you don’t know why the culture was taken in the first place and ya if it was just for urine is looking off and less likely for someone to have started it on call. Sometimes when it is started on call, the person kind of coming on is like, not sure why it was started and the communication bit is not that great there. So if there was a way to show that, that would probably help with the hospitalists as well.” (FG31)  Educational initiatives  “So we used the material from AMMI Canada, and they have a program, like a promotional material called ‘symptom free pee, let it be’ . So we put up the stop light system of posters around the floors that I was doing the project on and we have gotten quite a few comments and lot of feedback from the physicians and the nurses on it. That it has been helpful.” (FG14)  “I think more education for everyone involved with the types of things that you were talking about: when is it appropriate to test for, when it’s appropriate to treat.” (FG32) |
| 1. Social influences   (Those interpersonal processes that can cause individuals to change their thoughts, feelings, or behaviours)   - Social pressure or norms, Group conformity, Social comparisons, Group norms, Social support, Power, Intergroup conflict, Alienation, Group identity, Modelling | **To what extend do social influences facilitate or hinder X?**  Families wanting antibiotics or catheters in place  Pressure or recommendations from care teams  Influence of other healthcare providers or organizations, feedback from external groups influencing change  Comparison of performance between peers or groups (audit and feedback)  Feedback from other healthcare providers on prescribing or antibiotic use to individual healthcare providers or teams  Communication and teamwork | Patient/caregiver  “you might also see a decreased length of stay really in medicine because families are hesitant to send somebody home that are still on antibiotics, so if you started them when you didn’t need them then they say I am not taking them home for 3 more days until their antibiotics are done.” (FG15)  “a family is demanding something it is far easier to just give it to them instead of just taking the time to explain why they don’t need it. So in the case of asymptomatic bacteriuria it is easier to just prescribe an antibiotic and walk away instead of taking the time to have a talk.” (FG46)  “You know you have 6 daughters all want to come in a talk to you about why you are not going to treat the asymptomatic bacteriuria.” (FG54)  Healthcare professionals  “it’s an old culture thing cuz I was told by a senior nurse that these are the 2 things you look for, if they are positive, you have a UTI” (FG12)  “I would imagine if you have an established relationship with the physician, you feel comfortable sending it before having their OK, that you would probably say I will get their OK later.” (FG14)  “or the nursing homes- they are like, oh this is the exact same presentation as what they presented as last time. And was diagnosed as a UTI, so I think they have a UTI” (FG32)  “I have noticed there are times if we order, say we have someone who has SIRS, they are septic, they are very unwell, maybe it is not clear source, they get pip/tazo ordered as an example, I have had pharmacy come up to the floor and find me 24 hours later and say hey, that person is still on pip/taz, its been 24 hours, do you have a source identified yet? As part of the antimicrobial stewardship, which is awesome.” (FG54)  “having a catheter in verses when I order scheduled voiding Q4H it’s more resistance on a nurses time or a caregivers time.”  (FG52)  Audit and Feedback  “I think it would great to have a report card for facilities, a hospital thing, but also a prescriber specific, or team specific. On the medical unit the number of inappropriate urinalysis tests has decreased by 20%. Like I think that is important for us to know, cuz it’s good feedback to know I should be cognizant when I am doing this sample or sending the sample away. Cuz our unit has been doing very well.” (FG22)  “It would be good for us to know because not all these are generalizable that are done. So in your own particular unit, how many times did I as a physician treat a patient for a UTI who may have had a alternate explanation that was just a valid. How often I am doing that because that would just sort of reinforce. And I think even auditing around duration of antibiotics and things like that. So am I ordering 7 days of Cipro when I should be ordering 3, am I ordering. You know just that kind of thing because there really isn’t anyone checking on that.” (FG54) |
| 1. Emotion   (A complex reaction pattern, involving experiential, behavioural, and physiological elements, by which the individual attempts to deal with a personally significant matter or event)   - Fear, Anxiety, Affect, Stress, Depression, Positive/negative affect, Burn-out | **Does doing X evoke a physical response?**  Fear of getting worse or fear of consequences | “I think that is why everyone is so scared to not treat if they don’t have symptoms because it’s like what if it turns into something more and we didn’t start treating.” (FG13)  “Especially with everything that I work with, if your patient is on antibiotics and you’re like OK, they are covered. If anything happens they are covered.” (FG13)  “you are not going to get sued if you give somebody antibiotics and they didn’t need it, but you are going to get sued if you didn’t give antibiotics” (FG12)  “I feel like there is not as much anxiety if you have a plan and if your plan is – well I think it’s asymptomatic and we will monitor them for symptoms and if things change I’ll take the regular course.” (FG52) |
| 1. Behavioural Regulations   (Anything aimed at managing or changing objectively observed or measured actions)   - Self-monitoring - Breaking habit - Action planning | **Do you have systems in place to monitor whether you carried out X?**  Automatic urine cultures being sent on PPOs or through internal processes  Requiring staff to call for release of culture results  A mechanism to automatically flag durations for further review  Automatic stop orders | “I think that what they did in Moncton by withholding the culture results is like really spectacular.” (FG14)  “it is our policy in this institution to have 7 day stops for antibiotics, so it forces them to reassess. So that does generally happen.” (FG21)  “I haven’t seen it as much at this hospital, but at my last hospital, a urine culture was on the stroke protocol, for absolutely no reason. And the number of patients that came back with positive urines was obnoxious. So removing it from protocols would be a huge help. Unless there is some reason to do it, some indication that the patients is giving you but it is standard on that and it blew my mind.” (FG33) |

UTI = Urinary Tract Infection, ASB = asymptomatic bacteriuria, PPO – Pre-printed order

**Reference**

1. Cane J, O’Connor D, Michie S. Validation of the theoretical domains framework for use in behaviour change and implementation research. Implementation Science. 2012;7;(37):1-17.
